# ‘The burden is very much on yourself’: A qualitative study to understand the illness and treatment burden of hearing loss across the life course

**DOI:** 10.1111/hex.14067

**Published:** 2024-05-07

**Authors:** Sian K. Smith, Helen Pryce, Georgina Burns O'Connell, Saira Hussain, Rachel Shaw, Jean Straus

**Affiliations:** ^1^ Department of Audiology, College of Health and Life Sciences Aston University Birmingham UK; ^2^ Aston Institute of Health and Neurodevelopment and School of Psychology, College of Health and Life Sciences Aston University Birmingham UK

**Keywords:** audiology, hearing aids, hearing loss, illness burden, life course perspective, qualitative, treatment burden

## Abstract

**Introduction:**

Hearing loss is a chronic health condition that rises sharply with age. The way people respond to and cope with health conditions is influenced by their capacity to perform illness and treatment‐related work. The aim was to explore the cumulative burdens of living with hearing loss and the resources mobilised to ease the burdens.

**Methods:**

A qualitative design was used with semi‐structured interviews (online or in‐person) with participants recruited through audiology services and nonclinical services, such as lip‐reading classes. Forty‐six participants with hearing loss aged between 16 and 96 years were interviewed. An abductive approach, informed by May et al.'s burden of treatment theory, was used to analyse the data.

**Results:**

The illness burden involved participants working to make sense of their hearing loss, engaging in emotional work in response to changes in sound, social interactions and identity and coping with the daily frustrations required to communicate with others. Abandonment and uncertainty characterised the treatment burden; participants engaged in emotional work to adjust to hearing technology and deal with the uncertainty of how their hearing might progress. To ameliorate the burdens, participants drew on internal resources (psychological, health literacy, cognitive) and external resources (social support, financial, information, technology).

**Conclusions:**

The workload of hearing loss appears largely devolved to the patient and is not always visible. Our work indicates the need to widen approaches in audiological care through the implementation of lifeworld‐led care, family‐centred care and peer support to build support for those with hearing loss.

**Patient or Public Contribution:**

We developed the project in consultation with members of the public who have lived experience of hearing loss recruited through Aston University and volunteer links to audiology services. We also consulted people more likely to be affected by hearing loss adults including adults with learning disabilities, older adults in residential care and people from South Asia (Bangladeshi, Indian and Pakistani communities). These individuals commented on the study aims, interview schedule and participant recruitment practices. One of our co‐authors (expert by experience) contributed to the development and interpretation of themes and preparation of the final manuscript.

## 1 INTRODUCTION

Hearing loss is a global health concern affecting around 1.5 billion people (one in five) worldwide.^1^, ^2^, ^3^ By 2050, the numbers affected are expected to rise to 2.45 billion (one in four) (Haile et al., 2021). As we age, hearing loss increases, and is the third highest cause of disability worldwide (Haile et al., 2021). Hearing loss is a long‐term condition that can have significant and far‐reaching implications, adversely affecting communication, educational attainment, psychosocial wellbeing and quality of life.^4^, ^5^ Many older people with hearing loss also live with multiple co‐existing conditions that require different management strategies. Hearing loss is associated with arthritis, cancer, cardiovascular risk factors, diabetes, stroke and visual impairment,^6^ and is a modifiable risk factor for dementia.^7^

Patients are increasingly expected to manage their chronic conditions alongside everyday life demands. Living with a long‐term health condition involves work, such as learning how to identify and manage symptoms, dealing with negative thoughts and coordinating care (organising appointments and managing medication). There are concerns that this work places demands on patients and their families rather than supporting a shared responsibility of care model.^8^ As health conditions accumulate over time, the increasing demands of managing multiple conditions may exceed a person's capacity. Capacity refers to the resources (affective, cognitive, informational, material, physical and relational) that can affect a person's ability to perform the work and tasks of chronic illness.^9^, ^10^ An imbalance of workload and capacity can negatively impact quality of life, wellbeing and healthcare outcomes.^11^, ^12^ Understanding a person's capacity to cope with hearing loss within the broader context of their lives is important in addressing the burden and ensuring the workload is manageable.^10^

Several models have sought to conceptualise the workload of living with chronic health conditions. Corbin and Strauss propose three types of work needed to manage chronic illness, namely: illness‐related work (e.g., preventing and managing symptoms), everyday life work (e.g., managing the demands of the household) and biographical work (e.g., how a person reconstructs their identity and life goals).^13^ Another important model—The Burden of Treatment Theory—is a useful framework for understanding the interplay between the workload transferred to patients and their families (social networks) by healthcare systems and their capacity to manage it.^12^ This model considers burdens of *illness* (e.g., coping with hearing loss) and burdens of *treatment* (e.g., accessing and living with interventions). The extent to which patients and their support networks engage in illness and treatment work is contingent on *functional performance* (cognitive and material capacity), *social skills* (being able to engage and negotiate the assistance of others), *social capital* (access to informational and material resources) and *structural resilience* (the extent to which support networks can buffer adversities).^12^

The illness burden of hearing loss can be significant. Hearing loss adversely affects communication, resulting in individuals withdrawing from, or avoiding social situations due to listening fatigue (increased cognitive efforts to listen, manifesting in mental exhaustion) and frustration.^14^, ^15^ To optimise communication, affected individuals report using visual cues (e.g., lip‐reading), nonverbal cues (e.g., facial expressions) and asking others to speak clearly, slow down and repeat information.^15^, ^16^

Seeking help with hearing is linked to a sense of responsibility for communication difficulties.^17^ When hearing aids are prescribed, the treatment workload entails patients getting used to distorted amplified sounds, coping with the perceived stigma and managing devices.^18^ Hearing aids do not completely eradicate communication difficulties experienced (e.g., listening in background noise), and may divert attention from the psychosocial implications.^19^ In addition to the treatment workload, patients may not be ready for this treatment and benefit from sharing their feelings.^17^ The notion of ‘lifeworld‐led care’—exploring an individual's sociocultural background, beliefs and priorities within their lifeworld—could provide a useful approach when exploring the hidden work of hearing loss.^20^, ^21^

It is important to gain insight into the cumulative workload of hearing loss from the patient's perspective. Clinicians may not be aware of the patient's workload and capacity. The current study sought to explore the characteristics of the burdens of *illness* and *treatment* experienced by people living with hearing loss across the life course, and the *resources* they draw upon to manage the workload. This work forms part of a larger study—the Hearing Loss and Patient Experience study (HeLP study)—the first study internationally to develop a Patient Reported Experience Measure (PREM) to understand the daily burdens of hearing loss (illness and treatment work experienced by patients) to help tailor audiology care.^22^, ^23^

## 2 MATERIALS AND METHODS

### 2.1 Patient and public involvement engagement (PPIE)

We developed the project in consultation with PPIE groups recruited through Aston University and volunteer links to audiology services. We also targeted groups more likely to be affected by hearing loss, namely adults with learning disabilities, older adults in residential care and people from South Asia (Bangladeshi, Indian and Pakistani communities). Our PPIE groups advised on recruitment and checked analysis interpretations.

### 2.2 Recruitment and sampling

Potential participants (young people and adults aged over 16 years old with lived experience of hearing loss) were recruited through clinical services in the South West of England (Bath and Bristol) and Scotland (Tayside), and nonclinical routes, such as lip‐reading classes and residential care homes. Our clinical sites were chosen to provide a contrast in location (rural, urban and semi‐urban) and socioeconomic variation. Audiology staff informed patients about the study and posters were displayed in the waiting areas and public toilets. Details were advertised on social media and the university website. Interested participants directly contacted the study team via email or telephone and were provided with further information. All participants gave written informed consent. A total of 46 participants aged between 16 and 96 years were interviewed, of which 29 were female, 34 wore hearing aids, 20 were recruited from clinical sites and 26 through nonclinical routes (Table 1). We recruited from seven regions (South West and Midlands, North‐East, South‐East and mid‐Scotland) including postcode districts with affluent and low‐income communities.

**Table 1:** Sample characteristics.

Characteristic	*N*
Age group	
16–29 years	6
30–49 years	13
50–79 years	14
80 years–end of life	6
Gender	
Female	29
Male	17
Hearing aid user	
Yes	34
No	12
Recruited from clinical sites	20
Recruited via non‐clinical routes	26

### 2.3 Data collection

During 2022–2023, semi‐structured interviews were conducted by four female qualitative researchers. This included two academic‐clinician researchers (a clinical scientist [SH], a hearing therapist [H P]) and two academic researchers with a sociology background (GB OC) and health psychology background (SS). Interviews were either conducted online using Microsoft Teams or at the participants' preferred location (at home, the university or the audiology department). Open‐ended questions explored emotions about hearing loss, daily challenges, decisions around help‐seeking and experiences with audiology services (Table 2). With participants' consent, the interviews were audio‐recorded using a digital voice recorder or Microsoft Teams. Field notes were taken to note information not captured by the recordings. In‐person interviews were transcribed verbatim by a professional transcription service; online interviews were transcribed using Microsoft Teams transcription and checked for accuracy by the researcher. Transcripts were anonymised with all identifying information removed.

**Table 2:** Interview questions.

Tell me a little bit about yourself? Tell me your story with your hearing and why you were interested in taking part? Can you tell me your thoughts and feelings about hearing? What have you found difficult about having a hearing? What have you done to manage your hearing loss? Decision making around hearing aid use or non‐use/uptake—tell me how you came to be using/not using your assistive listening devices/hearing aids and reasons why/why not? What is important to you when deciding whether to use hearing devices/aids? Who else is important in helping you make decisions? What has been helpful to you? Have you sought help from audiology services? Tell me about your experience with using audiology services. Based on your experience—what do you think they ought to know/to do that they currently do not do?

### 2.4 Data analysis

The data were analysed thematically by the burden of treatment theory^12^ to understand how participants described the ‘work’ of living with hearing loss and the resources they drew upon to ease the burdens. Abductive reasoning was used in this phase to critically appraise the themes identified.^24^, ^25^ Our analysis involved researchers (HP, GBOC, SH, SS) reading transcribed interviews to identify examples of ‘work’ and ‘resources’ within participants' accounts. Next, we labelled examples of the types of work and resources described by participants, and then grouped these labels into key themes, denoting the different features of the ‘illness work’, ‘treatment work’ and ‘resources’ that people used, and noted differences across the life course.

## 3 RESULTS

### 3.1 Qualitative findings

**Figure 1:**
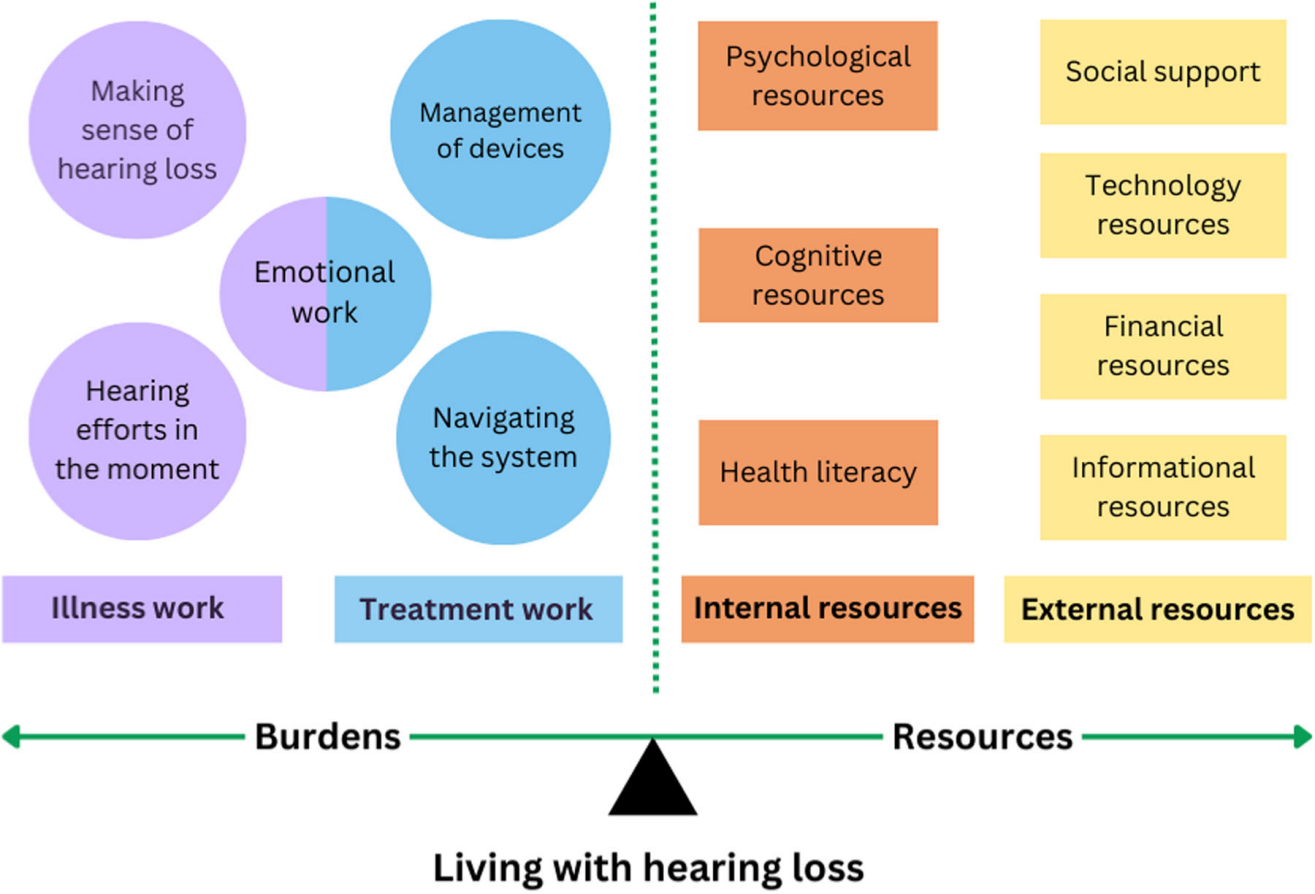
Diagram representing the burdens (illness and treatment work) and resources accessed and/or experienced by individuals living with hearing loss.

The illness burden of hearing loss appeared to be threefold as participants: (1) worked to *make sense of their hearing loss* (e.g., symptoms, cause, type, severity), (2) engaged in *emotional work* in response to changes in sound, communication, identity and relationships and (3) described *hearing efforts (cognitive and emotional) at the moment* when communicating with others (Figure 1).

Treatment work was characterised by participants: (1) *navigating the health system*, which did not always consider the communication needs of a person with hearing loss nor signpost to relevant services (e.g., peer support, hearing therapy) and (2) *managing hearing technology/devices* and undertaking *emotional work* to cope with uncertainty.

The burdens of hearing loss were alleviated through a sense of agency in participants, influenced by their *psychological resources* (e.g., resilience, personal growth, confidence), *cognitive resources* (problem‐solving, concentration, perception) and *health literacy skills* (acquiring knowledge, applying critical appraisal skills). External resources (*social*, *technology*, *financial* and *information*) also shaped participant's capacity to manage hearing loss.

It was striking how participants spoke more about the burdens of hearing loss (illness and treatment work) than their capacity (resources, skills). Interviewers needed to delve deeper to understand the participant's capacity. The results have been organised in accordance with participants' accounts with the ‘burdens’ representing the key themes (albeit resources weaved into each theme). Differences between age groups are indicated; otherwise, there are no differences.

#### 3.1.1 Making sense of hearing loss: Past, present and future

The illness work began with participants developing awareness of their hearing loss through recognition of their hearing difficulties in different listening situations. Participants tapped into their cognitive resources (social cognition, attention, perception) to construct symptoms of hearing loss. For many, this realisation often stemmed from problems they had with comprehending and conversing with others (e.g., not being able to hear grandchildren, asking people to repeat themselves, missing information).

I couldn't hear what other people were saying at meetings, even though the people either side of me had no problems. (Participant 15; age range 50–79)

Participants also reflected on their hearing performance in comparison to others and from observations and remarks made by family and friends regarding how they acted (e.g., increasing TV volume, mishearing speech, not hearing the doorbell). For some, views from others about their hearing reinforced their own suspicions about their hearing and prompted them to seek help. For others, comments appeared difficult to accept and their readiness to seek help was not immediate; however, this changed when participants perceived there to be a decline in their hearing which affected themselves and their relationship with others.

For some years my family said that I was deaf. So, in the end I gave up and had a hearing test. (Participant 33; age range 80+)

The work of treatment involved, in part, understanding audiometry testing procedures, interventions and technical language. Participants had to make sense of audiograms and get up to speed with medical terminology and phrases. Participants' health literacy skills enhanced their capacity to interpret and apply their knowledge to make decisions about their hearing. The process of clinical help‐seeking was, however, not always straightforward, and most participants spoke of needing to be proactive and self‐motivated to secure appointments and access audiology services.

It took from June until about 3 weeks ago (6 months in total) to get an appointment. That's energy and time out of my life. (Participant 46; age range 30–49)

Across all age groups, participants spent time and effort making sense of what had happened with their hearing and were searching for answers as to the cause. They reflected on incidents in childhood and adolescence (e.g., ear infections, accidents), their family histories and other potential causes (e.g., noise exposure, age). Participants tapped into several resources (cognitive, health literacy, social, informational) to process and assimilate information and (re)construct their auditory reality. For example, one participant reflected on an incident in childhood, which they felt started a cascade of events leading to their hearing decline. Another participant felt their hearing loss was a combination of noise exposure in the workplace and genetics.

There was a certain amount of damage (from occupational hearing loss). I also inherited as my mother was deaf. (Participant 36, age range 50–79)

Reflecting on the past was juxtaposed with contemplating how their hearing loss might progress in the future. Some participants were preparing for the future and spoke of what they would implement should their hearing loss change (e.g., get a hearing dog, use a whiteboard to communicate with others at home, learn sign language). For some, there was concern about the genetic nature of their hearing loss and the likelihood of their children inheriting hearing loss.

It's always worried me that my child will develop a hearing impairment as he gets older cause that's what happened to me. (Participant 21; age range 30–49)

Participants wanted clarity about how their hearing might progress in the future. Dealing with this ambiguity left participants feeling frustrated and anxious. Participants created their own capacity to cope with the uncertainty by drawing on internal psychosocial resources (resilience, religious faith, fatalism attitudes, emotional support and social comparison).

My faith (in God) is what gets me through and knowing that there's always someone in a much worse off position than yourself. (Participant 43; age range 30–49)

#### 3.1.2 Hearing efforts in the moment

Regardless of age, participants described the additional efforts they made to socially connect with other people. When conversing with others, hearing loss (and the work they needed to do) appeared to be at the forefront of participants' minds and somewhat inescapable during interactions. Cognitive resources were required to concentrate and pay attention to visual and non‐verbal cues (e.g., facial expression, lips, body language) to keep up with conversations. Additional work was also needed to verify information (e.g., asking others to repeat information, checking the topic, seeking the assistance of others) and to be aware of one's surroundings (e.g., strategically moving to face the person speaking to have good light).

I said to the waitress, ‘Can we move?’ They had a bit of natural light coming in and I said, ‘Can we sit over there?’ (Participant 32; age range 50–79)

Due to the increased listening efforts, mental exhaustion was a common feeling experienced by participants. To cope with the exhaustion, participants craved silence, took periods of rest (including short naps) and removed their hearing aids to help switch off and restore energy.

The exhaustion isn't anywhere near as bad as it used to be when I had to go to an office. I was absolutely wrecked at the end of every single day. (Participant 31; age range 50–79)

To cope in social situations, participants developed skills (self‐advocacy, patience, resilience) and spent time and labour on educating others about hearing loss. Not only did they prompt and remind others how to communicate during ‘in the moment’ situations but they also rectified (mis)assumptions that people had about hearing loss and hearing aids, which were often connected with stigma and discrimination (e.g., people misjudging them as rude, misconceptions around their intelligence and cognitive ability, age, ability to work or perform in a role).

Having the confidence to be able to sit there and admit that you can't hear them and you're not being rude. (Participant 26, age range 16–29)

Efforts were made to convince others of their hearing loss by increasing its visibility or clarifying judgements.

You get a comment like, ‘You don't look deaf.’ I say, ‘How am I meant to look? I've got hearing aids’. (Participant 36; age range 50–79)

Participants often found group conversations with background noise more work than one‐to‐one conversations in a quiet environment. Participants expressed frustration when people spoke quietly and appeared to struggle in more formal situations, such as work meetings, where there may have been shame in disclosing hearing loss and asking for adaptations. The illness work involved spending time anticipating upcoming social situations and foreseeing their control over acoustical environments. Participants drew on their cognitive resources to, for example, plan where they might sit, order food in advance and organise assistive devices. Participants also anticipated how others might respond to their hearing loss.

I assess what accommodation I need to cope better in that situation—do I come out as someone with hearing loss or just cope? (Participant 17; age range 30–49)

Instrumental support from family and friends enhanced participants' capacity to manage the illness workload. Participants valued it when other people made efforts to reduce their workload during conversations, including facing the person directly, speaking clearly, calling the person's name to get attention, not talking in another room, remembering if a person hears better in one ear and position accordingly, choosing a meeting place with minimal background noise, making sure the person is aware of the topic of conversation and avoiding rapid changes in topic. Participants described instances where family and friends forgot to make accommodations contributing to feelings of frustration and causing tension and discord in relationships.

My wife has a habit of jumping subjects. I can be completely lost and I'm frequently saying, ‘I haven't got a clue what you're talking about’. (Participant 38; age range 80+)

Participants' capacity for self‐advocacy about their hearing loss increased over time with experience.

It's only been in the last 10 years I've been able to stand in the post office and say, ‘I've asked three times for you to find out how to switch on the induction loop’. (Participant 31; age range 50–79)

### 3.2 Navigating the system

Accessing care was also complicated by the perceived lack of communication between departments (e.g., audiology, ENT, general practitioners) regarding the patient's medical files. Participants were often tasked with the burden of repeating information about their hearing condition to multiple healthcare professionals.

I thought there was a system in place to access hospital records from different hospitals. So, I'm having to explain everything to each appointment. (Participant 43; age range 30–49)

Participants' health literacy skills enhanced their capacity to confidently communicate with different healthcare professionals and coordinate their audiology care. In some cases, the complexity of the health system could lead participants to feel as if all their resources (time, energy, relational, cognitive) were required to navigate the system. Participants emphasised the importance of empathetic healthcare professionals who listened to them.

The current crop of audiologists, they're just such nice people. They are empathetic and have a degree of understanding about what this hearing loss must be like to live with. (Participant 31; age range 50–79)

Extra work for participants also came from the perceived lack of awareness and empathy in health systems (including audiology services) about the communication barriers and lack of adaptations for people with hearing loss. While waiting for appointments, participants described not hearing the buzzer or their name being called. One participant described making a name sign to ensure she did not miss her appointment.

### 3.3 Management of devices

Hearing aids appeared to be the most common intervention offered to participants; there seemed to be limited knowledge of other options available beyond hearing aids. In a few cases, participants applied their health literacy skills to research other options (e.g., assistive listening devices, lip‐reading, hearing therapy).

I asked about (hearing therapy) some time ago and then talking to the hearing therapist who ran lip‐reading classes. (Participant 39; age range 50–79)

Treatment work was characterised by challenges such as acclimatisation to hearing aids, fitting aids, replacing batteries and tubing, keeping aids dry and clean, wearing hearing aids and glasses and evaluating hearing aid performance. Although acclimatising to the hearing aids could be challenging, some described them as ‘life‐changing’ and valued the role they played in supporting the illness work (e.g., enabling participants to engage in small group conversations).

Having hearing aids has allowed me to continue living my life, mostly as it was before. (Participant 47; age range 30–49)

The instrumental support that participants (mostly adolescents and those aged over 80 years) received from family and friends enhanced their capacity to manage their treatment workload. Such tangible support included organising and accompanying patients to audiology appointments, negotiating with clinicians (on the patients' behalf) and providing practical help (collecting batteries and attending hearing aid repair clinics).

My wife helps me with getting wax out of the tubing. (Participant 35; age range 80+)

More uncertainty arose from not knowing whether their hearing aids were performing at their optimum or whether their hearing loss was declining. The lack of clear‐cut answers and difficulties accessing National Health Service (NHS) audiology services led some participants to draw on their time, energy and financial resources to, for example, purchase batteries and access private audiology care as they perceived the private sector to have better quality hearing aids and shorter waiting times. Some participants also expressed frustration at the cessation of ear wax removal in the NHS and having to draw on financial resources to use private providers.

There's only so much they can do on the National Health which is free. That's the service you get. Unfortunately, if it's not good enough, you've got to go and get private, which I've done. (Participant 23; age range 80+)

#### 3.3.1 Emotional work

A consistent finding across the age groups was the social disconnection and isolation that participants experienced in relation to their hearing loss. They described feeling ‘left out’ and ‘cut off’ from conversations, which in turn created work for participants as they internalised and coped with several emotions (e.g., grief, loneliness, frustration, embarrassment).

I'm very lost in lots of people, and that makes you feel very alone and isolated. (Participant 37; age range 80+ years)

Emotional distress was alleviated through support provided by family and friends when they conveyed empathy and acted as advocates for participants in group situations. Sharing lived experiences of hearing loss through peer support (e.g., lip‐reading sessions) or receiving mutual support from partners/family members also experiencing hearing loss had the potential to reduce feelings of isolation. Note the use of pronoun ‘we’ in the quote below emphasising the importance of reciprocal support.

After church, people tend to talk, and my husband and I don't stay. We disappear or speak to them outside in the open. (Participant 15; age range 50–79)

Hearing loss affected the way participants viewed themselves and how they thought others viewed them. Psychological resources (resilience, acceptance, self‐reflection, social comparison) enabled participants to engage in illness work as they grappled with a (new) sense of identity. Often, participants described feeling ‘in the middle’, neither feeling part of the Deaf community nor feeling part of the hearing community.

I feel like the middle zone. There's a Deaf men's group, but I fear that I would feel like an idiot for turning up. I can't do sign language. (Participant 46; age range 30–49)

For younger participants (16–49 years), their sense of belonging seemed to diminish because very few, if any, of their peers were living with hearing loss.

It's kind of in limbo, where you don't know anyone your age. (Participant 21; age range 30–49)

By contrast, some older participants (age bands 50–79 and 80+ years) mentioned that hearing loss was common among their peers. Participants had spent time figuring out how to describe themselves (e.g., hard of hearing, deaf, hearing impaired). Some participants considered themselves a ‘fraud’ identifying as ‘deaf’ and even undeserving of clinical support in comparison to others who they perceived had greater hearing loss than themselves. A large amount of illness work involved contending with stigma (public and/or self‐stigma, internalised stigma) and discrimination linked to cognitive competence, agism and disablism.

It's all part of the stigma. You don't want to be seen as being old and decrepit. (Participant 23; age range 80+)

The word ‘acceptance’ was commonly used by participants—coming to terms with their hearing loss as part of their lives. The process of acceptance was associated with reconstructing a new normal.

I've accepted it. The hearing loss is not gonna come back and that's OK. Before I was always so negative. I'm now not ashamed to say that I've got hearing loss (Participant 26; age range 16–29 years)

For some participants, this process took time and was facilitated through growing self‐confidence, resilience, the desire to be socially connected, fatalistic attitudes and unwavering support from others. At the same time, participants expressed concern about becoming too reliant on others and not wanting to feel burdensome. Participants spoke of changing their life priorities. Such a shift was associated with psychological resources (personal growth and reflexivity) as demonstrated by a participant who decided to change careers.

Hearing loss impacted my confidence within my professional life. It had a real impact from that perspective that I've decided to completely change careers. (Participant 50; age range 30–49)

Taking part in the research afforded participants, albeit possibly for the first time, the opportunity to share their emotions, suggesting that coping with hearing loss had rested on their shoulders. Some participants drew on their own health literacy, time and financial resources to attend counselling.

Treatment work was not only characterised by the practical day‐to‐day management of hearing aids but also the emotional work of adjusting to wearing hearing aids and coping with distorted sound (‘echoing in my head’) and feeling ashamed. Participants also felt disappointed when their hearing aids did not meet their or others' expectations (i.e., the assumption that hearing would be resolved/normal/fixed).

Touching on the hearing aid point and being naive myself until the point of experiencing it. It's not some magic device that is gonna fix everything. (Participant 1; age range 30–49)

Additional emotional work arose from audiometry testing which generated trepidation among some participants. The apprehension before testing, the experience of being left by oneself (i.e., in a soundproof booth) to perform a test they felt ‘set up to fail’ and the fear of test results (which may show a decline in hearing) was distressing.

All my audiology appointments I dread because it is mentally draining and the hearing test in the boxes at the hospital it's like a little prison. You're not going to do your best. (Participant 26; age range 16–29)

## 4 DISCUSSION

This paper sought to understand the characteristics of illness and treatment burdens experienced by people living with hearing loss across the life course. Our work sheds light on the invisible work incurred by hearing loss outside of the clinical encounter.^26^ Irrespective of age, it seemed that participants were working hard to make sense of their hearing loss and what the auditory changes might mean for their sense of identity, interactions, relationships and future selves. They were also working hard to minimise communication breakdown with family and friends. This required increased cognitive effort (concentration and listening) and physical effort (positioning to hear), often resulting in mental exhaustion. Across the life course groups, the treatment workload may be made more onerous by poorly coordinated services, lack of communication adaptations for patients with hearing loss in audiology services, a sense of abandonment when acclimatising to hearing aids and treatment costs (e.g., wax removal, hearing aids from private providers).

Our analysis revealed a large amount of emotional work. The challenge of feeling socially disconnected, having a poor sense of deaf identity and experiencing stigma and emotions (e.g., sense of loss, frustration, loneliness, uncertainty) are all part of the emotional burden. While these are well established, we suggest they should be reframed as emotional work. In line with previous work, the emotional demands of treatment work, such as getting used to wearing hearing aids, new auditory sensations and implications for sense of self, were evident.^14^, ^27^, ^28^, ^29^ Less well‐researched is the psychological impact of audiometry testing and the onus on the individual to cope with feelings of anxiety. Emotional burden is rarely elucidated in the burden of treatment theory.^30^, ^31^

Our study contributes to a growing number of studies which propose that the emotional burden is a critical part of living with a chronic condition(s).^11^, ^31^, ^32^ The process of acceptance played an important role in helping participants reconstruct a new normal and sense of self about their hearing. This aligns with the concept of ‘biography’ depicted in the theory of patient capacity.^9^ Reframing one's biography in the face of adversity, can help individuals to create meaning in their lives and enhance their experience.^9^ Similarly, Bury's^33^ concept of ‘biographical disruption’ emphasises the disruption caused by chronic illness to a person's sense of self, and Corbin and Strauss' notion of ‘biographical work’ reflects the lifelong work that people engage in to reconstruct their identities.^13^, ^34^ Likewise, as our findings suggest recreating a new biography takes time and may not happen overnight. Coping mechanisms align with those found in previous research, including adopting fatalistic beliefs linked to religion/spirituality, downward social comparison and drawing on psychosocial resources (e.g., resilience, reflexivity, social support).^9^

For some, this process took time and was facilitated through growing self‐confidence, resilience, the desire to be socially connected, fatalistic attitudes and support from others. At the same time, participants expressed concern about relying on others and not wanting to feel burdensome. Participants spoke of changing their life priorities. Such a shift was associated with psychological resources (personal growth and reflexivity).

Our study highlights the importance of *relationality*—the social networks in which individual *agency* can be mobilised.^12^ Some participants drew on practical support from family and friends (*collective action*) to help them manage the treatment workload (e.g., assistance with hearing aids, accompanying them to appointments) and facilitate conversations with others. However, the experience of living with hearing loss appeared an individualised one in which the responsibility mostly devolved to the patient across different domains (e.g., employment, family life, healthcare).^35^ This resulted in participants feeling mentally exhausted and despondent.^15^, ^36^ Participants enlisted others to help them, which in turn boosted their social capital (their ability to access resources) and structural resilience (whereby others absorb adversity through mobilising resources).^12^ Peer support at lip‐reading classes and mutually supportive relationships (in which a family member or friend also had hearing loss) seemed important in enhancing social capital and structural resilience.^37^

Our findings also shed light on the different types of health literacy that participants drew on as they (i) acquired knowledge about their hearing loss (functional health literacy), (ii) interpreted and discussed information with significant others (communicative health literacy) and (iii) critically considered their options (critical health literacy).^38^, ^39^ Our data suggest that while participants engaged in functional and communicative health literacy, there were only a few who drew on advanced cognitive and social skills (critical health literacy) to consider options beyond hearing aids (e.g., lip reading, hearing therapy).

Although we recruited participants from regions with contrasting areas of affluence and poverty, we did not formally record socioeconomic or ethnic characteristics or existing chronic conditions. These characteristics are likely to influence participants' ability to engage with their social networks and access health services.^12^ We also note that it was difficult to recruit younger people, as reflected by the lower numbers in the youngest age group. This work was conducted in the aftermath of the coronavirus disease 20‐19 pandemic where challenges and changes to routine audiology practice have been observed.^40^

There are several important implications for clinical practice. First, the findings provide important insights into the illness and treatment work to manage hearing loss. Previous work shows that it can be difficult for patients, carers and healthcare professionals to view living with an illness and enacting treatment as ‘work’.^41^ Indeed, much of the work (particularly illness work) may be unseen by healthcare professionals. For example, the considerable effort needed for patients to listen, concentrate and (re)educate people about their hearing.

Second, it is important to be mindful of how patients' (and carers') capacity to manage tasks varies depending on the resources available.^9^, ^12^, ^42^ Individuals with fewer resources (e.g., poorer social support, lower financial resources, limited health literacy) and those managing additional chronic conditions are likely to struggle and have reduced capacity. The theory of patient capacity proposes five psychosocial mechanisms that can hamper or strengthen patient capacity: patient biography (Biography), their resources (Resources), their environment (Environment), their ability to accomplish life and patient work successfully (Work) and their social networks (Social), coined as (BREWS). This mnemonic may remind healthcare professionals to consider how well a patient is integrating their illness and its treatment into their lifeworld, what resources they can draw upon, how their environment and ability to undertake work (‘illness’ or ‘treatment’) affects their capacity and the social support they receive.^9^, ^43^ Resources are not static and may fluctuate daily. External (rather than internal) resources might be more susceptible to change because an individual has less control over these resources. For example, a lip‐reading class might stop due to funding and subsequently diminish an individual's social resources or the hearing induction loop may not be working properly (technology resource). Identifying ways to support and build patient capacity is important.^9^ Patients struggling with the emotional challenges of hearing loss might benefit from hearing therapy or group support.

Third, in line with existing research, participants reported communication barriers with audiology staff in the reception area and the importance of additional communication (e.g., visual screens to alert patients).^44^ A recent survey highlighted that assistive communication devices were not available in one‐third of audiology clinics in England. Staff were not familiar with this technology and/or had limited awareness of the communication barriers for people with hearing loss.^45^ Communication skills training for all audiology staff, including reception/administrative staff, is important to raise deaf awareness and enhance accessibility.

Finally, our findings underline the importance of healthcare professionals adopting a holistic approach to understand what it is really like to live with hearing loss. There has been growing recognition for person‐centred care in audiology, whereby patients are encouraged to express their preferences.^46^, ^47^ We propose that lifeworld‐led care, with its focus on the influence of cultural, environmental and social contexts in shaping health and wellbeing, has a valuable place in audiological rehabilitation.^20^, ^21^, ^38^ The lifeworld‐led approach has the potential to shift the focus of clinical encounters from hearing aid prescription to open up opportunities to understand what really matters to the individual in relation to their life circumstances and discuss other interventions (e.g., support groups, use of assistive devices).^48^, ^49^ Family‐centred care, shifting focus from the individual to a shared familial responsibility for communication, also warrants exploration in audiology.^50^, ^51^, ^52^

Moving forward, a key output for our own research is a novel Patient Reported Experience Measure (known as a PREM), which aims to capture the lived experience of hearing loss in a succinct, but comprehensive way.^22^, ^23^ It can be used by healthcare professionals to quickly identify how a patient is coping with hearing loss, understand the hidden work undertaken by patients and identify aspects of their hearing loss experience that could be supported. It is designed to complement (and potentially streamline) existing practice and record how patient experience evolves over time.

## 5 CONCLUSIONS

The experience of hearing loss takes significant effort, involving hidden practical, psychosocial and relational work that is devolved to the individual. Participants drew on several internal and external resources to learn about their hearing loss, communicate with and educate others, manage devices and cope with the residual uncertainty and feelings of disconnection inherent in the experience. It is important to be mindful of the workload and responsibility delegated to the individual and build patient capacity to ease burdens; widening approaches in audiological care (e.g., lifeworld‐led care, family‐centred care) could help to achieve this.

## AUTHOR CONTRIBUTIONS

**Sian K. Smith**: Project administration; investigation; visualisation; formal analysis; writing—original draft; methodology; writing—review and editing. **Helen Pryce**: Conceptualisation; funding acquisition; investigation; project administration; visualisation; supervision; methodology, formal analysis; writing—review and editing; **Georgina Burns O'Connell**: Project administration; investigation; methodology; visualisation; formal analysis; writing—review and editing. **Saira Hussain**: Investigation; project administration; methodology; formal analysis; writing—review and editing. **Rachel Shaw**: Conceptualisation; funding acquisition; supervision; methodology; formal analysis; writing—review and editing; methodology. **Jean Straus**: Visualisation; formal analysis; writing—review and editing.

## CONFLICT OF INTEREST STATEMENT

The authors declare no conflict of interest.

## ETHICS STATEMENT

Ethics approval was gained from the West of Scotland Research Ethics Service (approval date: 6 May 2022, ref: 22/WS/0057) and the Health Research Authority and Health and Care Research Wales (HCRW) Approval (approval date: 14 June 2022; IRAS project ID: 308816). Participants gave informed consent to participate in the study before taking part. Informed written consent was obtained from all participants involved in this study.

## Data Availability

The data that support the findings of this study are available on request from the corresponding author. The data are not publicly available due to privacy or ethical restrictions.
